# Neopterin extends *C. elegans* lifespan in an ATFS-1-dependent manner

**DOI:** 10.17912/micropub.biology.001543

**Published:** 2025-03-21

**Authors:** Miina Pitkänen, Geoffray Monteuuis, Christopher B. Jackson, Olli Matilainen

**Affiliations:** 1 The Molecular and Integrative Biosciences Research Programme, Faculty of Biological and Environmental Sciences, University of Helsinki, Helsinki, Uusimaa, Finland; 2 Department of Biochemistry and Developmental Biology, Faculty of Medicine, University of Helsinki, Helsinki, Uusimaa, Finland

## Abstract

Neopterin, a byproduct of tetrahydrobiopterin synthesis, is commonly used as a biomarker for immune system activation. In addition to its role in immune responses, neopterin levels are known to increase with age. Its impact on longevity, however, remains unclear. Here, we demonstrate that neopterin supplementation extends lifespan in *Caenorhabditis elegans .* Additionally, neopterin shows moderate activation of the mitochondrial unfolded protein response (UPR ^mt^ ), and that the neopterin-mediated lifespan extension is dependent on ATFS-1 , the primary transcription factor regulating UPR ^mt^ . These findings highlight the need for further investigation into the biological functions and health-promoting effects of neopterin.

**Figure 1. Neopterin supplementation extends the lifespan of C. elegans:**
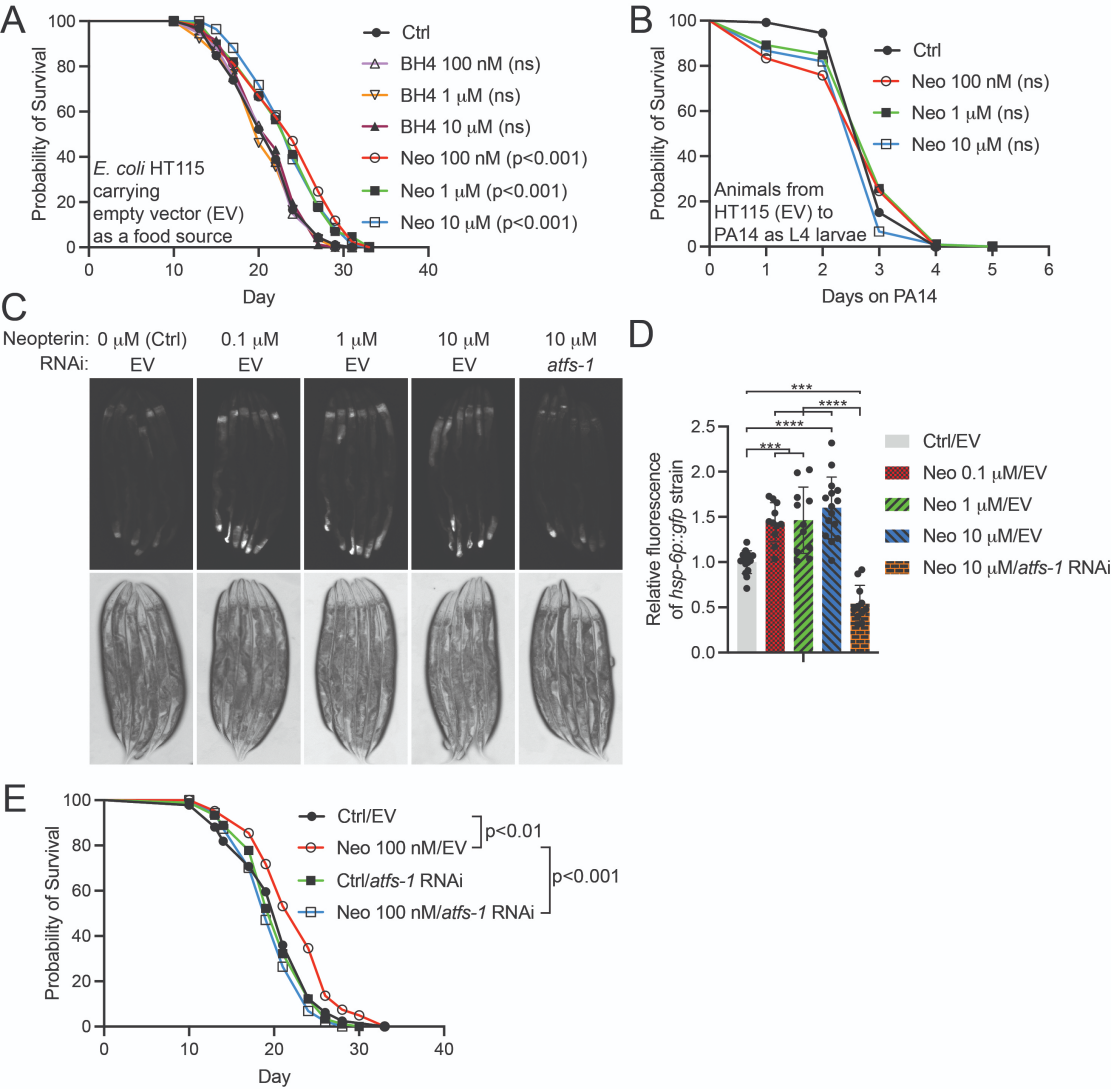
**Figure 1. A** ) Lifespan of wild-type *C. elegans* (N2) on plates containing indicated concentration of tetrahydrobiopterin (BH4) or neopterin (Neo), compared to control. *E. coli* HT115 carrying the empty vector (EV) was used as the food source (ns: not significant). **B** ) Survival of neopterin-treated *C. elegans* on *Pseudomonas aeruginosa* (PA14). **C** ) Images of neopterin- and EV or *atfs-1* RNAi-treated, day 1 adult UPR ^mt^ reporter strain ( *hsp-6p::GFP* ). **F** ) Quantification of fluorescence signal in neopterin-treated *hsp-6p::GFP* animals. Each dot represents a group of six animals. Data for Ctrl/EV and Neo 10 mM/EV are from four independent experiments (n = 90 animals), and for Neo 0.1 mM/EV, Neo 1 mM/EV, and Neo 10 mM/ *atfs-1* RNAi from three independent experiments (n = 66 animals) (***p < 0.001, ****p < 0.0001, one-way ANOVA with Tukey's test). **E** ) Lifespan of control- and neopterin-treated N2 animals on EV or *atfs-1* RNAi. Lifespan statistics are reported in Extended Data Table 1.

## Description

Neopterin (d-erythro-neopterin), a molecule belonging to the pterin group, was first isolated from bee larvae. Since its discovery in the 1960s, neopterin has been identified in a variety of organisms, including humans. Neopterin is produced by monocyte-derived macrophages and dendritic cells (Hoffmann et al., 2003), and its elevated levels in urine and blood have been used for decades as a marker of immune system activation in conditions of viral, bacterial, and parasitic infections, autoimmune diseases, cancer, neurological disorders, and cardiovascular diseases (Murr et al., 2002). Apart from circulating neopterin, it can be independently synthesized in the brain. This has been demonstrated by multiple studies showing that neopterin levels in plasma and cerebrospinal fluid (CSF) do not correlate in patients with inflammatory disorders (Dale et al., 2009; Kuehne et al., 2013; Millner et al., 1998; Molero-Luis et al., 2013).

Neopterin is a byproduct of the tetrahydrobiopterin (BH4) *de novo* synthesis pathway. BH4 is an endogenous cofactor required for enzymatic conversions of essential biomolecules, including nitric oxide and monoamine neurotransmitters (Eichwald et al., 2023). In this pathway, guanosine triphosphate (GTP) is converted to BH4 through the actions of GTP cyclohydrolase I (GTPCH, also known as GCH1), 6-pyruvoyl-tetrahydrobiopterin synthase (PTPS), and sepiapterin reductase (SPR). Additionally, non-specific reductases, such as aldose reductase (AR) and carbonyl reductase (CR), may also contribute to BH4 synthesis (Eichwald et al., 2023). Under normal conditions, GCH1 is the rate-limiting enzyme in BH4 synthesis (Werner et al., 2011). However, during immune system activation, interferon-gamma (IFN-γ) upregulates GCH1 activity, making PTPS the rate-limiting factor and leading to increased neopterin production (Müller et al., 1991; Schoedon et al., 1986; Werner et al., 1990). Notably, monocytes and macrophages have reduced PTPS activity, further favoring neopterin production in these cells (Leitner et al., 2003). In addition to IFN-γ, INF-a/b and endotoxins have also been shown to stimulate neopterin production (Bloom et al., 1990; Cano et al., 2008).

Interestingly, although neopterin is strongly linked with inflammation and immune system activation, it has been reported to inhibit inflammasome activation (De Paula Martins et al., 2018). Furthermore, neopterin has been shown to have atheroprotective effects (Shirai et al., 2018; Watanabe, 2021), to counteract hyper-inflammation and oxidative stress (Al-kuraishy et al., 2021), and to function as a cytoprotective molecule in the brain (Ghisoni et al., 2015, 2016). These findings suggest that, in addition to being a marker of inflammation, neopterin also has health-promoting effects.

Studies in humans, mandrills, and capuchins have found that neopterin exhibits a U-shaped relationship with age, with levels being elevated in both early and late life (Dibakou et al., 2020; Lucore et al., 2022; Werner et al., 1987). Studies in adult Barbary macaques and chimpanzees have shown that neopterin levels are elevated in older individuals (Müller et al., 2017; Negrey et al., 2021). Together, these reports indicate that the aging-associated increase in neopterin levels is a conserved process across species. Although neopterin levels fluctuate during aging and immune system activation, it remains unclear how this particular pterin affects lifespan. To investigate this, we utilized *Caenorhabditis elegans* as model. Importantly, endogenously produced neopterin is detected in *C. elegans* (Yin et al., 2020), supporting its use in experiments testing how neopterin influences an organism's physiology.

First, we investigated how neopterin and BH4 affect lifespan. Both metabolites were supplemented into the NGM agar at final concentrations of 100 nM, 1 μM, and 10 μM. Interestingly, while BH4 showed no effect on longevity, neopterin was found to increase lifespan ( Fig. 1A, Extended Data Table 1), suggesting that it has a protective role in the organism's physiology. In this study, we independently performed seven lifespan experiments with 100 nM, four experiments with 1 μM, and six experiments with 10 μM neopterin. Notably, 100 nM and 1 μM neopterin significantly extended lifespan in every experiment, whereas 10 μM neopterin did not lead to a significant change in lifespan in two independent experiments (Extended Data Table 1). These observations raised the question of whether neopterin becomes toxic at higher concentrations. To address this, we performed lifespan experiments using plates supplemented with 25 μM and 50 μM neopterin. These treatments did not significantly affect lifespan (Extended Data Table 1), demonstrating that neopterin is not toxic at the tested concentrations.

Since neopterin has been strongly associated with immune system activation (Murr et al., 2002), we investigated whether it modulates survival on pathogenic bacteria. To address this, we utilized a slow-killing assay with the pathogenic *Pseudomonas aeruginosa* strain PA14 (Tan et al., 1999). We performed three independent survival assays with neopterin-treated animals on PA14 . In these assays, animals were transferred from neopterin-containing plates (100 nM, 1 μM, and 10 μM) to PA14 plates at the L4 larval stage. Animals from the 100 nM and 1 μM neopterin plates showed significantly improved survival on PA14 in one experiment, whereas none of the tested neopterin concentrations affected survival in the other two experiments ( Fig. 1B, Extended Data Table 1). These data suggest that, whilst the tested neopterin concentrations extend lifespan ( Fig. 1A, Extended Data Table 1), they do not consistently affect survival on pathogenic bacteria.

Over the past decades, extensive research has shown that moderate activation of stress response pathways under normal conditions can lead to extended longevity (Soo et al., 2023). Based on this, we hypothesize that neopterin extends lifespan through a stress response-associated mechanism. One such mechanism is the mitochondrial unfolded protein response (UPRmt), which promotes mitochondrial homeostasis in response to stress (Shpilka & Haynes, 2018), and has been linked to longevity (Durieux et al., 2011; Houtkooper et al., 2013; Soo et al., 2023). Since neopterin has been shown to increase the number of mitochondria in sensory neurons (Eichwald et al., 2023) and to protect mitochondria from sodium azide-induced stress in cultured rat astrocytes—a treatment that also enhances neopterin release in these cells (Ghisoni et al., 2015)—we investigated whether neopterin activates the UPR ^mt^ . To test this, we utilized the *hsp-6p::gfp* reporter strain, which exhibits increased fluorescence upon mitochondrial stress (Yoneda et al., 2004). We found that all tested neopterin concentrations increase fluorescence intensity, particularly in the tail-end of the intestine ( Fig. 1C- D), suggesting that neopterin moderately activates UPR ^mt^ . Knockdown of *atfs-1* , the central regulator of UPR ^mt^ (Nargund et al., 2012), reduces fluorescence signal upon neopterin treatment ( Fig. 1C- D), indicating that neopterin activates this mitochondrial stress-induced transcription factor. Furthermore, *atfs-1* RNAi blocks the lifespan extension in animals treated with 100 nM or 10 μM neopterin ( Fig. 1E, Extended Data Table 1), further demonstrating that neopterin exerts its protective effect through ATFS-1 . Together, these data reveal an intriguing link between neopterin and lifespan and encourage further investigation into how this pterin affects health.

## Methods

**NGM plates for *C. elegans* experiments**

Except exposure to *Pseudomonas aeruginosa ,* *C. elegans* strains, N2 (wild type) and *hsp-6p::GFP* ( SJ4100 ), were maintained on NGM plates (peptone, P4963, Merck; agar, A4550, Merck; NaCl, 746398, Merck) seeded with *E. coli* HT115 bacteria carrying empty vector (EV, control vector for RNAi) or vector inducing *atfs-1* RNAi (clone from the Vidal RNAi library). Plates contained also ampicillin (100 mg/ml), tetracycline (10 mg/ml), and IPTG (0.5 mM). D-(+)-neopterin (Merck, N3386) was dissolved in DMSO to obtain a 10 mM stock solution. (6R)-5,6,7,8-Tetrahydrobiopterin dihydrochloride (Merck, T4425) was dissolved in H _2_ O to obtain a 10 mM stock solution. Both compounds were added to NGM media at indicated concentrations. The DMSO concentration was kept consistent across all experimental conditions.

***C. elegans* lifespan assays**

All lifespan experiments were performed at 20 ^o^ C on *E. coli* HT115 containing either the empty (EV)- or *atfs-1* RNAi vector. Bacteria were grown using the protocol described earlier (Timmons et al., 2001). Lifespan experiments were initiated by letting P0 generation hermaphrodites to lay eggs on NGM agar plates, and the F1 generation was scored for lifespan. At the L4 larval stage animals were transferred to plates containing 5-fluorouracil (10 µM) (Merck, F6627) to prevent progeny production. Animals were maintained on the indicated RNAi/neopterin/BH4 plates for their entire lifespan. Animals that had exploded vulva or that crawled off the plate were censored. Animals were counted as dead if there was no movement after poking with a platinum wire. Lifespans were checked every 1-3 days. Lifespan graphs were generated using GraphPad Prism (version 10.4.1 (532)). Statistical calculations for lifespan experiments were carried out in R Studio (version 2024.12.1+563) by using the Cox-proportional hazard regression analysis. Statistical details for the lifespan data can be found in Extended Data Table 1.

***Pseudomonas aeruginosa* assay**

*Pseudomonas aeruginosa* assays were performed as described earlier (Tan et al., 1999) with minor modifications. 3 cm NG plates supplemented with 5-fluorouracil (10 µM) (Merck, #F6627) were seeded with 3 µl of an overnight-grown *Pseudomonas aeruginosa* ( PA14 ) suspension and incubated at 37°C for 24 hours. 20 μl of 2% SDS was added to the edges of the plate to prevent the escape of the animals. *C. elegans* were grown on 6 cm NGM agar plates supplemented with indicated neopterin concentration. Animals were transferred to PA14 plates at the L4 stage and incubated at 25°C. Animals were scored daily for survival based on their ability to respond to touch. Animals that crawled off the plate were censored. The graph depicting survival on PA14 was generated using GraphPad Prism (version 10.4.1 (532)). Statistical calculations for the survival on PA14 were carried out in R Studio (version 2024.12.1+563) by using the Cox-proportional hazard regression analysis. Statistical details of PA14 experiments can be found in Extended Data Table 1.

**Imaging**

P0 generation hermaphrodites of *hsp-6p::GFP* ( SJ4100 ) animals were let to lay eggs on NGM agar plates with indicated neopterin concentration and RNAi bacteria, and the F1 generation was imaged as day 1 adults using a standard stereomicroscope with fluorescent light source (Zeiss). For the imaging, animals were placed on unseeded NGM-agar plate and immobilized using tetramisole hydrochloride (30mM) (Merck, L9756) diluted in M9 solution. Fluorescence intensities were quantified using Fiji (version 2.14.0/1.54f) (Schindelin et al., 2012). Statistical analyses and bar graph were generated using GraphPad Prism (version 10.4.1 (532)).

## Data Availability

Description: Extended Data contains Table 1, which shows statistics of individual lifespan experiments.. Resource Type: Dataset. DOI: https://doi.org/10.22002/1rrtn-3a029
